# A machine learning model utilizing CT radiomics features and peripheral blood inflammatory markers predicts the prognosis of patients with unresectable esophageal squamous cell carcinoma undergoing PD-1 inhibitor combined with concurrent chemoradiotherapy

**DOI:** 10.7150/jca.105171

**Published:** 2025-03-03

**Authors:** Xudong Liu, Fei Gao, Shusheng Wu, Haoyu Wang, Wenxi Dang, Mingjie Sun, Zhihua Zhang, Mengge Li, Zhirun Cai, Wen Li, Yifu He

**Affiliations:** 1 Wannan Medical College, Wuhu, Anhui, 241002, China.; 2 The First Affiliated Hospital of University of Science and Technology of China, Hefei, Anhui, 230031, China.; 3 University of Science and Technology of China, Hefei, Anhui, 230026, China.; 4 Anhui Medical University, Hefei, Anhui, 230001, China.

**Keywords:** immunotherapy, radiomics, markers of inflammation, nomogram

## Abstract

**Objective:** To investigate the value of a machine learning model that integrates radiomics features and peripheral blood inflammatory markers in predicting the prognosis of patients with unresectable esophageal squamous cell carcinoma (ESCC) receiving PD-1 inhibitor combined with concurrent chemoradiotherapy.

**Methods:** A retrospective collection was conducted involving 105 patients with unresectable ESSC who received PD-1 inhibitors combined with concurrent chemoradiotherapy at the First Affiliated Hospital of the University of Science and Technology of China from January 2020 to August 2023. These patients were randomly divided into a training set (n=74) and a validation set (n=31). Radiomics features were extracted from arterial phase CT images obtained before initial treatment, with feature selection performed using Pearson Correlation and LASSO-COX methods. Baseline clinical characteristics were analyzed, and hematological parameters were collected before the start of immunotherapy and within 4-6 weeks post-treatment to calculate inflammatory markers. Subsequently, independent radiomics features influencing patient prognosis were identified using a multivariate Cox proportional hazards model, and these features were incorporated into a clinical feature-based multivariate Cox model to derive independent prognostic factors combining radiomics and clinical characteristics. Nomograms were constructed to predict the 2-year progression-free survival (PFS) of patients based on the results of COX analysis involving clinical characteristics, radiomic features, and combined indicators. The models were evaluated and assessed using ROC curves and calibration curves.

**Results:** In the training cohort, the AUC was 0.705 for the clinical model, 0.573 for the radiomics model, and 0.834 for the combined model. In the validation cohort, the AUC was 0.784 for the clinical model, 0.775 for the radiomics model, and 0.872 for the combined model.

**Conclusion:** The combined model integrating the radiomic feature NGTDM-busyness, the inflammatory marker ΔNLR, and the clinical characteristic M stage offers the optimal predictive value for the 2-year PFS in patients.

## Introduction

Esophageal cancer ranks as the seventh most common malignancy and the sixth leading cause of cancer-related mortality worldwide 1. In China, esophageal squamous cell carcinoma (ESCC) is the predominant histological subtype, accounting for over 90% of all esophageal cancer cases 2. Due to the typically insidious onset of esophageal cancer, more than one-third of patients are already at an advanced stage at initial diagnosis, rendering them ineligible for curative surgery; these patients are considered unresectable 3. Unresectable ESCC patients can be categorized into two groups: locally advanced and metastatic, with differing standard treatment strategies for each. Concurrent chemoradiotherapy is the widely accepted treatment for locally advanced ESCC, while platinum-based systemic chemotherapy, with or without radiotherapy, is the main first-line treatment for metastatic ESCC 4. In recent years, the rapid advancement of immunotherapy has significantly altered the treatment landscape for unresectable ESCC. Monoclonal antibodies targeting programmed death-1 (PD-1) or programmed death-ligand 1 (PD-L1) have demonstrated compelling responses and clinical benefits across various malignancies, including ESCC 5. Multiple studies have confirmed that immunotherapy combined with concurrent chemoradiotherapy offers a significant survival advantage over definitive concurrent chemoradiotherapy alone in prolonging overall survival for patients with locally advanced ESCC 6-8. For metastatic esophageal cancer, immunotherapy combined with chemoradiotherapy has also become the current standard first-line treatment 9.

However, due to the heterogeneity of unresectable ESCC, there is significant variability in individual responses to immunotherapy in clinical practice 10. Although PD-L1 expression status, tumor mutational burden (TMB), and microsatellite instability (MSI) are currently recognized molecular markers, they have not yet been deemed ideal predictive markers 11. The selection of optimal patient subgroups and the search for effective predictive biomarkers have become hot topics in the field of immunotherapy. Previous studies have indicated that the immune microenvironment, through its interaction with immune cells, directly influences the efficacy of immunotherapy 12. In recent years, radiomics, a process that converts medical images into high-dimensional, minable, and quantifiable imaging features through high-throughput data extraction algorithms, has shown significant value in assessing the immune microenvironment and predicting responses to immunotherapy 13. Moreover, neutrophils, lymphocytes, monocytes, and platelets, as vital components of the immune microenvironment, constitute a comprehensive index of peripheral blood inflammatory markers derived from the aforementioned inflammatory cells, exhibiting outstanding efficacy in differentiating patient responses to immunotherapy across various solid tumors 14-16. The predictive value of models combining radiomics features and peripheral blood inflammatory markers for immunotherapy efficacy has been validated in non-small cell lung cancer 17; however, such studies have not yet been reported in esophageal cancer.

In this study, we extracted radiomics features from contrast-enhanced CT scans taken before the patients received their first treatment and established a radiomics model. Additionally, we analyzed the baseline clinical characteristics of the patients before treatment and extracted hematological parameters from before the first immunotherapy session and within 4-6 weeks after immunotherapy to calculate inflammatory markers. A clinical model was then constructed by integrating clinical characteristics and inflammatory markers. Finally, a combined model was developed by fitting radiomics features with inflammatory markers. The aim was to evaluate the predictive value of these three models for the 2-year progression-free survival of patients with unresectable ESCC receiving PD-1 inhibitor combined with concurrent chemoradiotherapy.

## Materials and Methods

### Study design and patients

**Figure 1:**
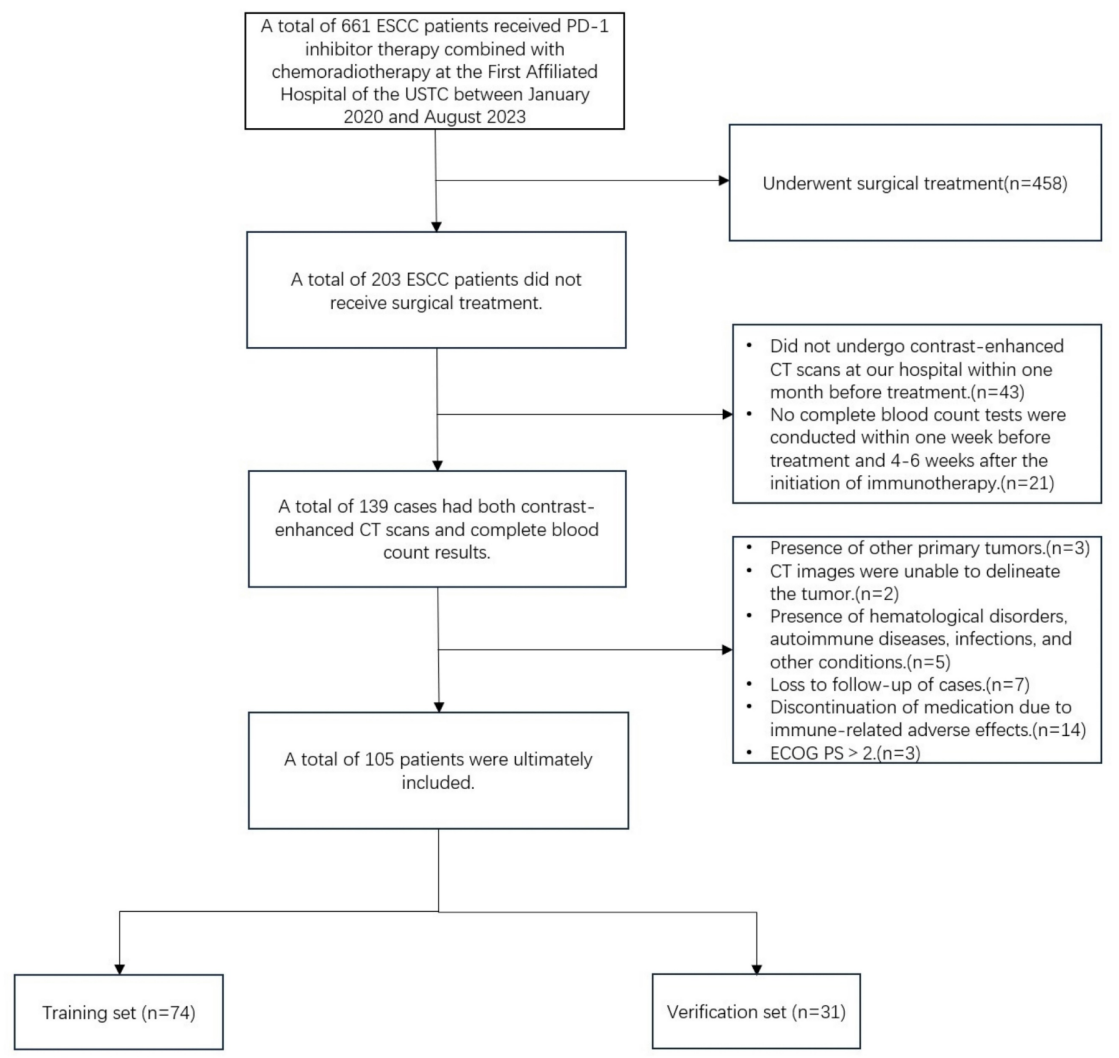
Patients enrollment flowchart.

This study retrospectively collected data from 105 patients with ESSC who received anti-PD-1 monoclonal antibody combined with chemoradiotherapy at the First Affiliated Hospital of the University of Science and Technology of China between January 2020 and August 2023. The follow-up period extended until March 1, 2024. All patients provided informed consent upon enrollment. This study complies with the Declaration of Helsinki (2013 version) and was approved by the Ethics Committee of the First Affiliated Hospital of the University of Science and Technology of China (approval number: 2024-ZNY-04). Inclusion criteria were as follows: (1) Histopathology confirmed squamous cell carcinoma; (2) Microsatellite stability (MSS) or proficient mismatch repair (pMMR); (3) clinical stages cT3-4N0M0/cT1-4N+M0 or cM1 (non-regional lymph node metastasis, radiotherapy during immunotherapy) according to the 8th edition of the AJCC; (4) enhanced CT scan performed within one month prior to the initiation of treatment; (5) completion of a complete blood count within one week before and 4-6 weeks after immunotherapy initiation; (6) Eastern Cooperative Oncology Group Performance Status (ECOG PS) ≤2; (7) receipt of three-dimensional conformal radiotherapy (3D-CRT) or intensity-modulated radiotherapy (IMRT), with at least two cycles of immunotherapy and at least one efficacy evaluation during treatment. Exclusion criteria included: (1) patients who underwent surgical treatment; (2) CT images in which the primary tumor could not be detected or segmented, or poor image quality; (3) presence of hematological disorders, autoimmune diseases, infections, or other conditions that could influence inflammatory markers; (4) loss to follow-up or discontinuation of immunotherapy due to severe immune-related adverse effects. Based on the study design for the predictive model, all patients were randomly divided into a training set and a validation set in a 7:3 ratio (Figure 1).

### Clinical characteristics

The clinical characteristics of the patients were collected, including age, gender, tumor location, TNM stage, treatment modality, radiotherapy technique, and chemotherapy regimen. Additionally, the absolute neutrophil count (ANC), absolute lymphocyte count (ALC), absolute platelet and monocyte counts, as well as albumin levels (g/L), were recorded within one week prior to the first immunotherapy session and 4-6 weeks post-immunotherapy. NLR was calculated as the ratio of ANC and ALC, PLR as platelet counts to ALC ratio, and SII as the absolute platelet count multiplied by NLR. MLR was calculated as the absolute monocyte count/ALC; albumin level (g/L) + 5 × ALC was defined as PNI. Additionally, the relative changes in NLR, PLR, SII, MLR, and PNI pre- and post-treatment were defined as the ratio between the corresponding time points and the markers pre-treatment, referred to as ΔNLR, ΔPLR, ΔSII, ΔMLR, and ΔPNI, respectively. The tumor location and clinical TNM staging were assessed using pre-treatment imaging modalities such as CT, PET-CT, and MRI. All staging was independently performed by two radiologists with over 15 years of clinical diagnostic experience, and any discrepancies were resolved through consultation. Patients underwent one of four distinct treatment regimens: Immune Checkpoint Inhibitor-Induced Chemoradiotherapy (ICI-CCRT), Concurrent Chemoradiotherapy with Immune Checkpoint Inhibitor (CCRT-ICI), Immune Checkpoint Inhibitor-Induced Chemoradiotherapy with Consolidation Immune Checkpoint Inhibitor (ICI-CCRT-ICI), Immune Checkpoint Inhibitor with Sequential Chemoradiotherapy (ICI-SCRT). The immunotherapy administered to all patients consisted of PD-1 inhibitors (pembrolizumab, nivolumab, tislelizumab, camrelizumab, sintilimab, toripalimab), and the chemotherapy regimen was platinum-based first-line therapy, including platinum combined with taxanes (TP), fluorouracil (PF), or docetaxel (DP), with dosages administered according to the recommended guidelines every three weeks.

### Scanning methodology

All patients underwent contrast-enhanced chest and full abdominal CT scans within one month prior to treatment, utilizing a 128-slice spiral CT scanner (Neusoft Medical NeuViz 128). Patients were required to fast for at least 6 hours before the examination, which was conducted in the supine position. The scanning range extended from the first cervical vertebra (C1) to the bilateral anterior superior iliac spines. The parameters for the contrast-enhanced CT scan were as follows: tube voltage of 120 kVp, tube current of 250-350 mAs, slice thickness of 5 mm, reconstruction interval of 5 mm, field of view of 35-50 cm, matrix size of 512×512, rotation time of 0.7 s, and a pitch of 1.375, using a standard reconstruction algorithm. Following the non-contrast CT scan, iopamidol (300 mg I/ml) was intravenously injected at a rate of 3.0 ml/s via a high-pressure injector at a dosage of 1.2 ml/kg. Arterial, venous, and delayed phase images were acquired at 30 seconds, 70 seconds, and 2 minutes after the initiation of contrast injection, respectively.

### Feature extraction and selection

**Figure 2:**
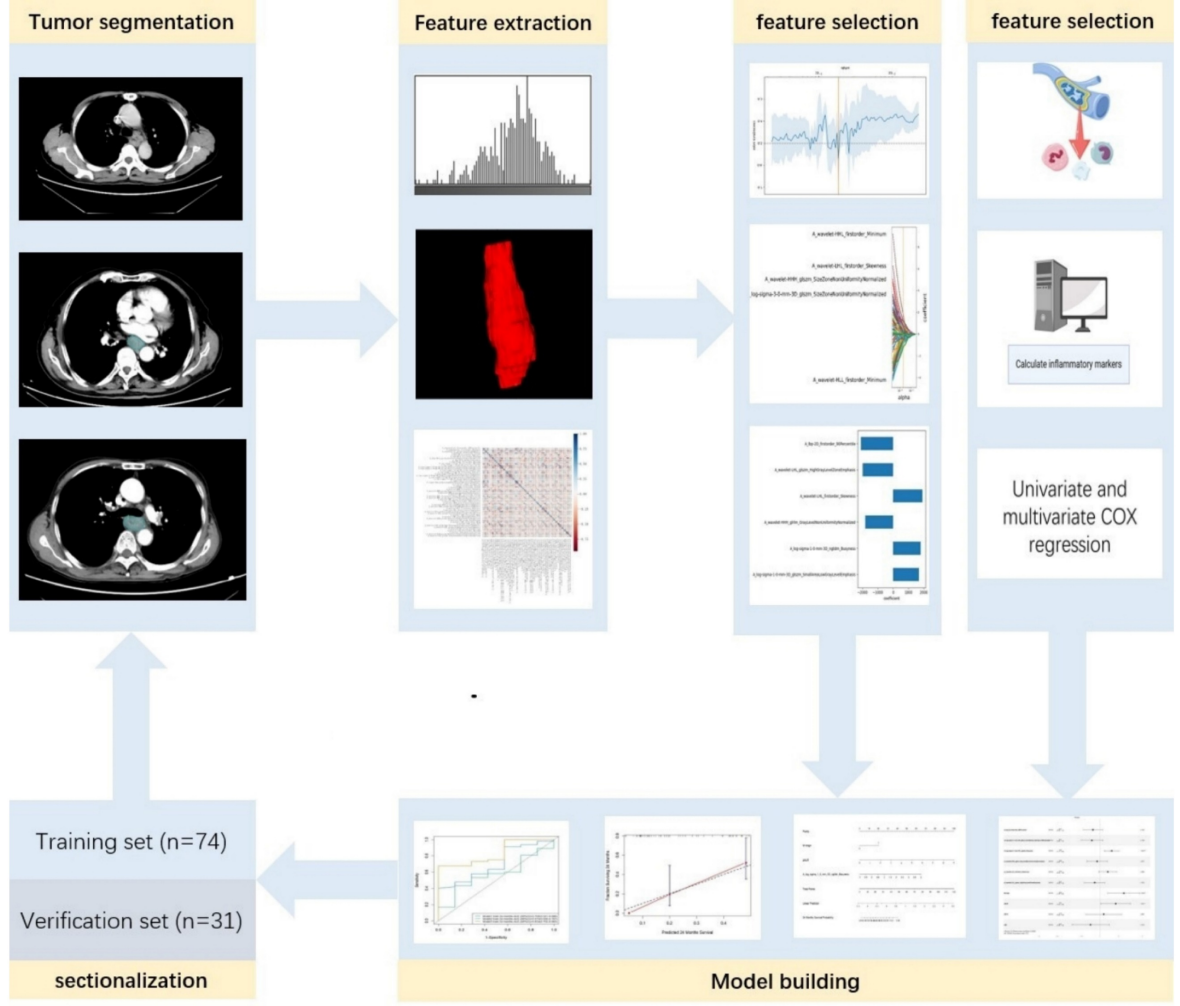
Radiomics workflow diagram.

The enhanced CT images were imported into ITK-SNAP (www.itk-snap.org), where the target lesion margins were delineated layer by layer on the arterial phase images, and subsequently merged into a three-dimensional region of interest. Two radiologists, each with over 15 years of clinical diagnostic experience, independently reviewed the images to ensure the reproducibility of the segmentation both within and between observers. The intra-observer agreement and inter-observer agreement of feature extraction were evaluated by correlation coefficients (ICCs). To compute the intra-observer ICC, 50 CT images were selected randomly and segmented twice in 1 month (at least 10 days apart) by reader A. To compute the inter-observer ICC, the selected images were segmented by two radiologists independently (reader A and reader B). Segmentation was performed to further obtain independent feature extraction to compute the intra-observer and inter-observer ICCs. When the ICC was greater than 0.75, it was considered good agreement, and the remaining segmentation was performed by reader A. The imaging histological features were extracted from the liver lesions using the Python-based PyRadiomics software package (http://pyradiomics.readthedocs.io), following the guidelines set by the Imaging Biomarker Standardization Initiative. Detailed explanations of these radiomic features can be found in the PyRadiomics documentation (https://pyradiomics.readthedocs.io/en/latest/index.html). Feature selection was performed using R software v. 4.3.2 (The R Foundation for Statistical Computing, Vienna, Austria). The Z-score normalization method was applied to standardize the scales of different features, ensuring that outdated feature scales were distributed within the range of 0 to 1. The "findCorrelation" function from the "caret" package in R was utilized to perform pairwise correlation analysis, with an absolute correlation cutoff set at 0.9, to eliminate redundant radiomic features. Subsequently, the Least Absolute Shrinkage and Selection Operator (LASSO) Cox regression was employed, which is a qualified method for regressing high-dimensional predictors by shrinking some regression coefficients to zero through penalization, to select the most predictive radiomic features from the training cohort. The penalty parameter (lambda) was determined through ten-fold cross-validation based on the minimum error criterion. The radiomics workflow is illustrated in Figure 2.

### Efficacy evaluation and study endpoints

Treatment response was assessed according to the [Response Evaluation Criteria in Solid Tumors (RECIST) v. 1.1] and categorized based on clinical data and imaging findings from electronic medical records into progressive disease (PD), stable disease (SD), partial response (PR), and complete response (CR). The primary endpoint of this study was progression-free survival (PFS), defined as the time from the initiation of anti-cancer therapy to the first documented progression, death, or the last follow-up.

### Model development and evaluation

The selected features from the training set were weighted according to the coefficients obtained from LASSO and were linearly combined to calculate the Rad-score. The cutoff value for the Rad-score was determined using X-tile software (version 3.6.1; Yale University School of Medicine, New Haven, USA), and patients were stratified into high-risk and low-risk groups based on this cutoff. Survival differences between the two groups were compared using the Kaplan-Meier method to preliminarily assess the association between the radiomics model and PFS. Previous studies have demonstrated that the predictive performance of radiomic features with high potential is significantly diminished when combined with an increased number of features, compared to when they are used independently 18. Therefore, following previous studies, a multivariate Cox proportional hazards model was used to select radiomic features (p<0.05) for inclusion in the subsequent model development 19, 20. The selected radiomic features were incorporated into a multivariate Cox proportional hazards model based on clinical characteristics to identify independent prognostic factors that include both radiomic and clinical features. Nomograms and calibration curves were constructed for the clinical model, radiomic model, and combined model. The predictive value of the three models for patient outcomes was assessed using the area under the receiver operating characteristic (ROC) curve and concordance index (C-index) in both the training and validation cohorts.

### Statistical analysis

Statistical analyses were conducted using R 4.3.2 and SPSS v. 27 (IBM Corp, Armonk, NY, USA). Descriptive statistics were performed for all variables. Normality tests were conducted for continuous variables. Data that followed a normal distribution were expressed as 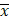
±s and compared between groups using independent samples t-tests. Non-normally distributed data were presented as M (Q1, Q3) and compared between groups using Mann-Whitney U tests. chi-square tests were used for categorical variables. A p-value of less than 0.05 was considered statistically significant. We used the “glmnet” package to perform the LASSOCox regression. The “rms” package was used for multivariable Cox regression analysis, nomogram construction and calibration. The “DynNom” package was used to build nomograms on the web. The R function cox.zph was employed to test the proportional hazards assumption for a Cox regression model fit. The C-index was calculated and compared using function concordance.index and C-index. comp in the “survcomp” package. Prediction error curves were generated using “pec” package.

## Results

### Baseline clinical characteristics

**Figure 3:**
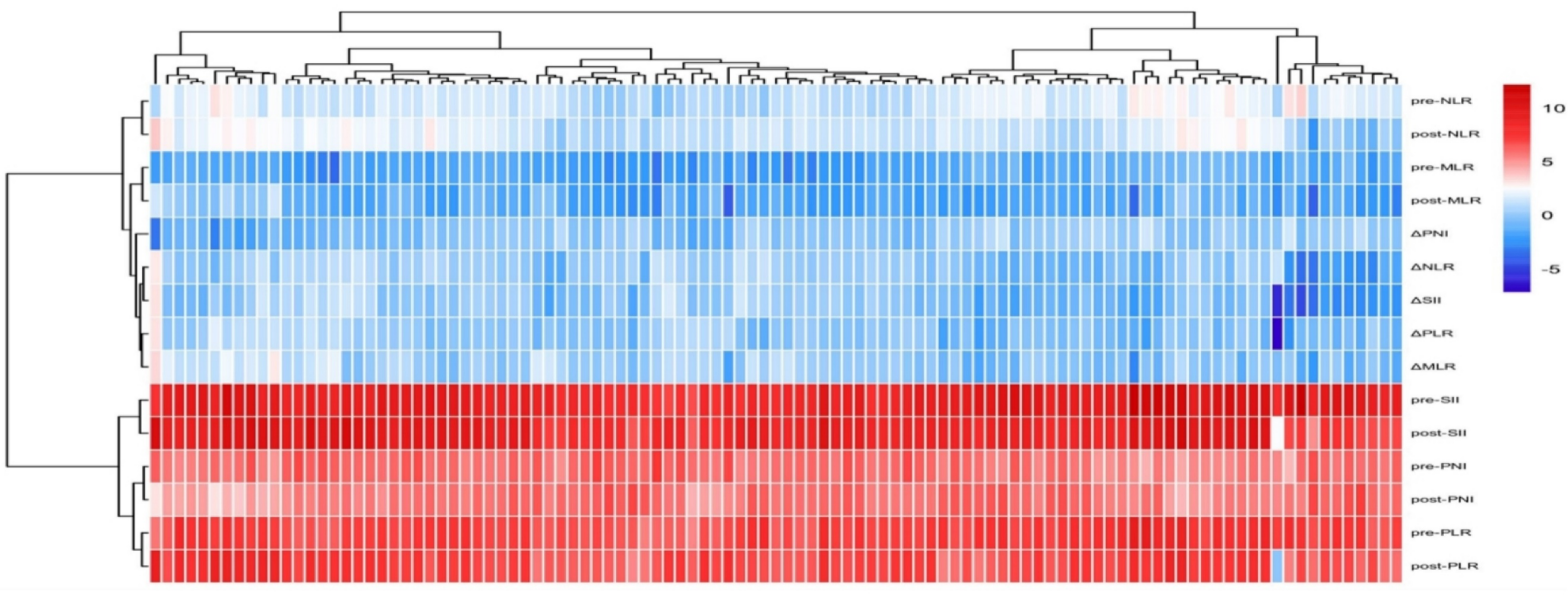
Heatmap of patient inflammatory marker distribution and clustering.

A total of 105 patients were included in this study, The median PFS for all patients was 12.30 months. The patients' ages ranged from 46 to 95 years, with a mean age of 67.34 ± 9.17 years. Among them, 4 patients (3.8%) achieved complete response (CR), 36 patients (34.3%) had partial response (PR), 54 patients (51.4%) experienced stable disease (SD), and 11 patients (10.5%) had progressive disease (PD). The disease control rate (DCR) was 89.5%, and the objective response rate (ORR) was 38.1%. The patients were randomly divided into a training set (n=74) and a validation set (n=31) in a 7:3 ratio. Baseline clinical characteristics of patients in the training and validation sets are shown in Table 1. Inflammatory markers for all patients are presented in Table 2, and their distribution is illustrated in Figure 3. Results indicated that, except for pre-treatment NLR and post-treatment NLR, no other clinical characteristics showed statistically significant differences between the training and validation sets.

### Reproducibility of radiomic features

Based on observer agreement both within and between observers, the ICCs for Reader A's two measurements ranged from 0.831 to 0.922. The interobserver ICCs between the two readers ranged from 0.736 to 0.907. These results indicate good reproducibility of feature extraction both within and between observers. Ultimately, all results were based on measurements by Reader A.

### Radiomic feature extraction

**Figure 4:**
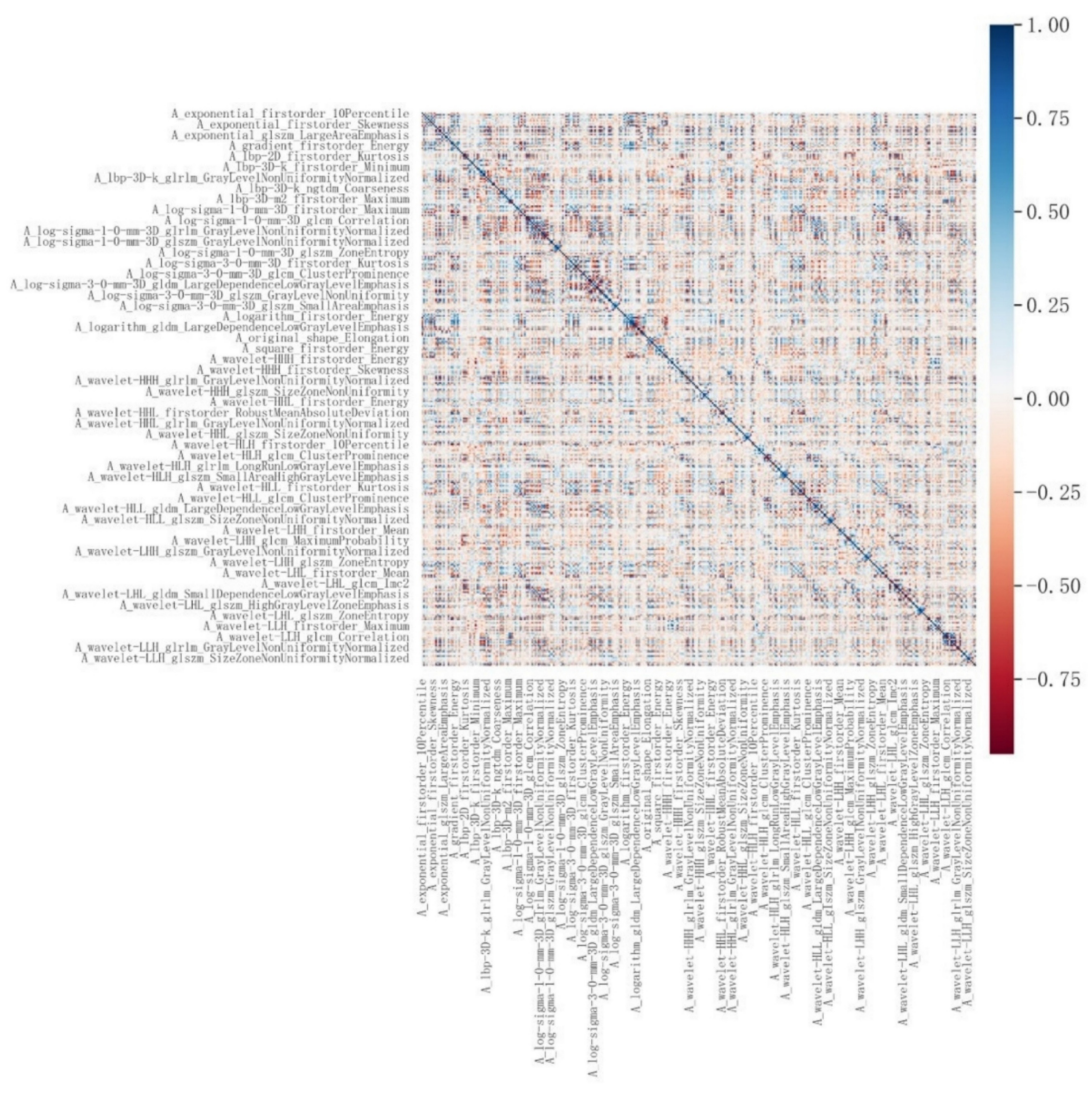
Correlation analysis of radiomic features.

**Figure 5:**
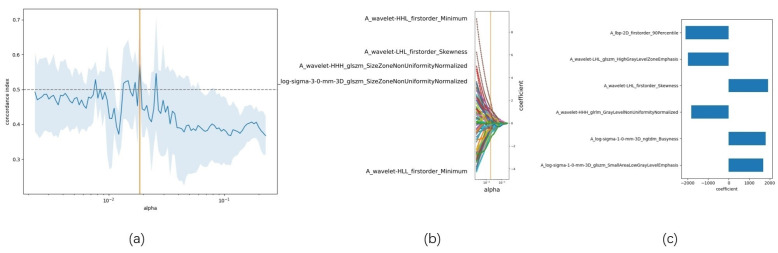
LASSO-based radiomic feature selection process. Note: (a) LASSO model accuracy score plot; (b) LASSO path diagram; (c) LASSO model feature weight plot.

A total of 1,874 radiomic features were extracted from the three-dimensional ROIs of esophageal cancer images (Figure 4). The original features included 14 shape features, 18 first-order features, and 75 second-order (texture) features (24 gray level co-occurrence matrix [GLCM] features, 14 gray level dependence matrix features, 16 gray level run length matrix [GLRLM] features, 16 gray level size zone matrix features, 5 neighborhood gray tone difference matrix [NGTDM] features). The original CT images were filtered using wavelet, Laplacian-of-Gaussian, square, square root, logarithm, exponential, gradient filters, Local Binary Pattern 2D/3D to generate higher-order statistics. Wavelet filters were applied using high (H) or low (L) pass filters to decompose the three-dimensional images, resulting in eight decompositions: HHH, LLL, HHL, HLL, LHH, LHL, LLH, and HLH. Local Binary Pattern 3D was employed to resample the images in three directions (Lbp-3D-k, Lbp-3D-m1, Lbp-3D-m2). Thus, the total number of radiomic features was 1,874 [(18 + 75) × 19 + 18 + 75 + 14 = 1874]. After excluding features with an absolute Pearson correlation coefficient ≥0.9, 363 features remained. Following dimensionality reduction using LASSO COX, a total of 6 radiomic features were identified (Figure 5, Table 3).

### Calculation of Rad-score

The Rad-score was calculated by weighting the selected features from the training set according to their respective coefficients obtained from LASSO. The formula for calculating the Rad-score is as follows:

Rad-score = -2099.693104633284*A_lbp-2D_firstorder_90Percentile +1674.37581167117771*A_log-sigma-1-0-3D_glszm_SmallAreaLowGrayLevelEmphasis +1790.137956984716*A_log-sigma-1-0-mm-3D_ngtdm_Busyness -1827.292786384293*A_wavelet-HHH_glrlm_GrayLevelNonUniformityNormalized +1914.8264558730364*A_wavelet-LHL_firstorder_Skewness -1991.3853589220475*A_wavelet-LHL_glszm_HighGrayLevelZone

**Figure 6:**
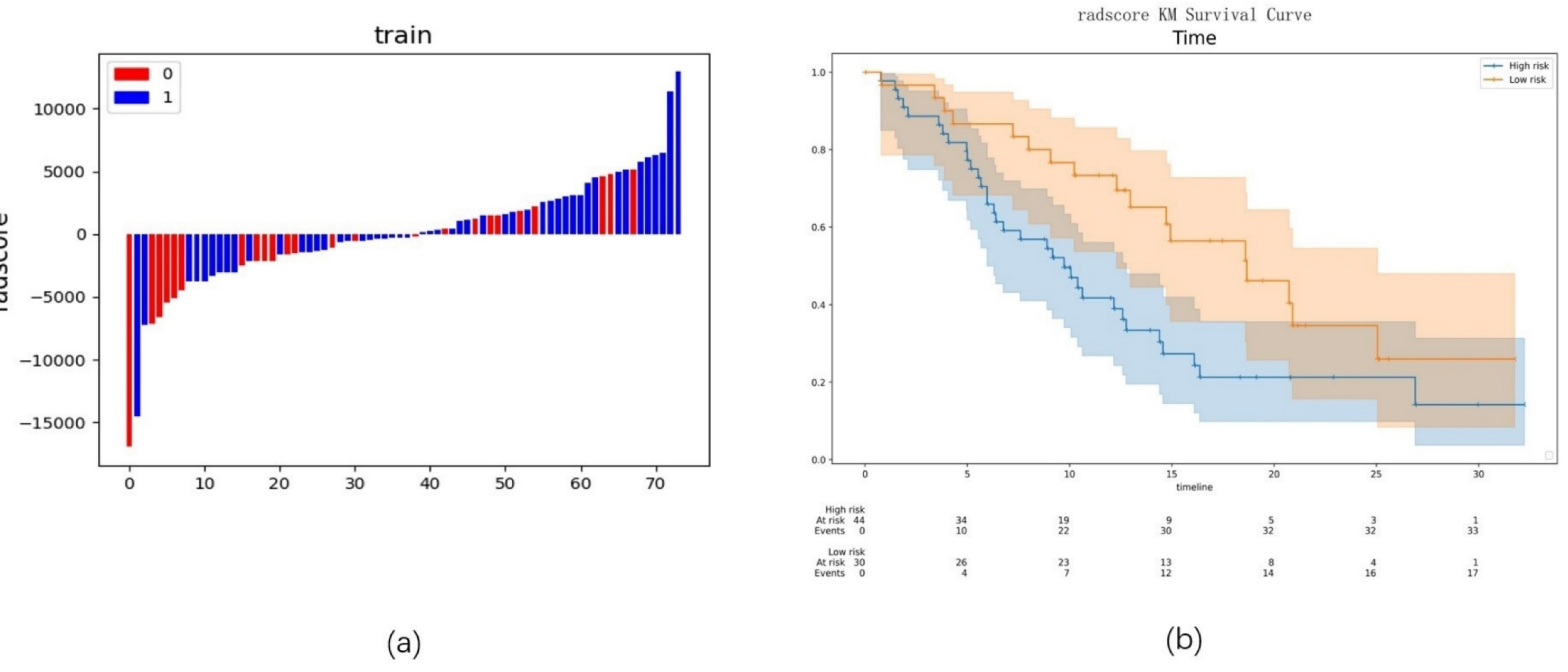
** Preliminary evaluation of the radiomics model.** Note: (a) Radiomics scores for each patient in the training group; (b) Kaplan-Meier survival analysis between low-risk and high-risk groups.

The cutoff value for Rad-score obtained using X-tile software was -561.77429933223. Based on this cutoff, patients were stratified into high-risk and low-risk groups (Figure 6a). Kaplan-Meier survival analysis revealed that the PFS in the low-risk group was significantly higher than that in the high-risk group (18.60 vs. 9.17, p=0.021) (Figure 6b).

### Selection of clinical and radiomic features

**Figure 7:**
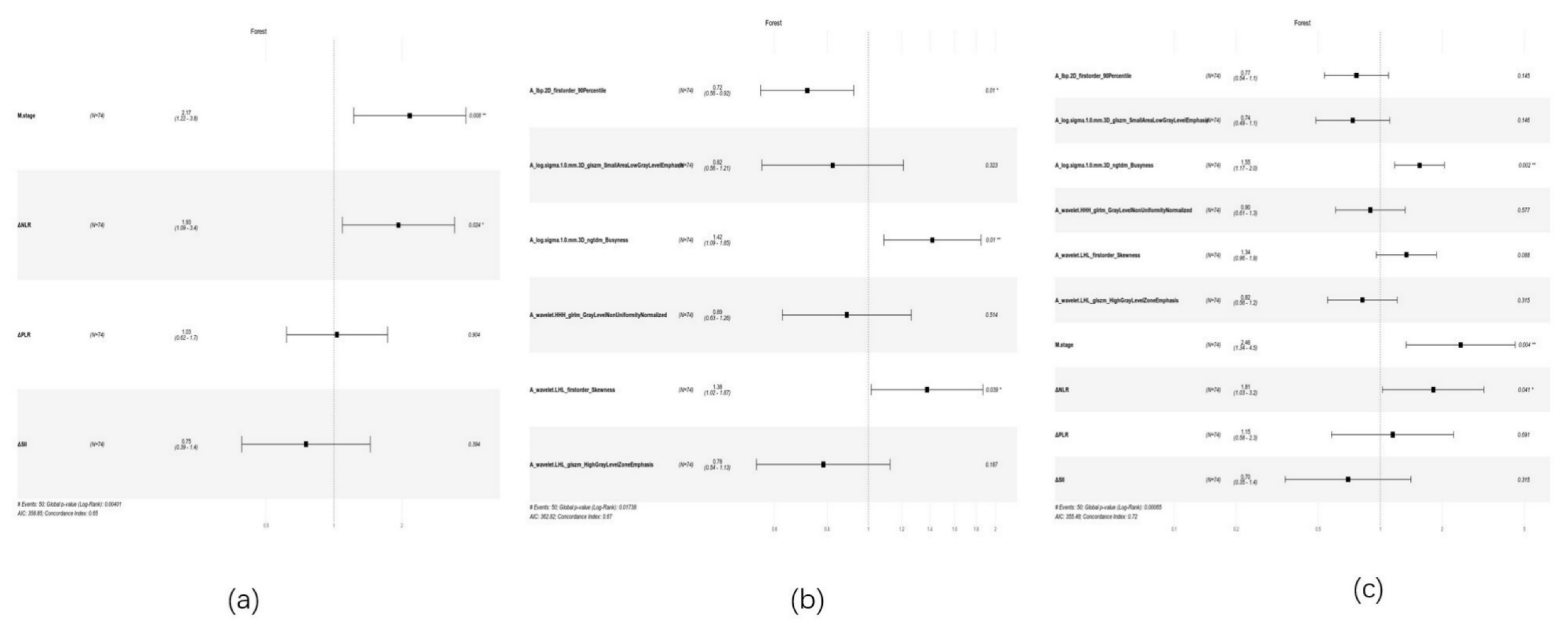
** forest plot of cox multivariate analysis.** Note: (a) Forest plot of Cox multivariate analysis for the clinical model; (b) Forest plot of Cox multivariate analysis for the radiomics model; (c) Forest plot of Cox multivariate analysis for the combined model

Univariate and multivariate Cox proportional hazards models were used to select clinical and radiomic features. Multivariate analysis of clinical features identified M stage (P=0.008, HR: 2.166, 95% CI: 1.222-3.840) and ΔNLR (P=0.024, HR: 0.176, 95% CI: 1.090-3.431) as independent risk factors affecting patient prognosis (Table 4, Figure 7a) (Figure s1). Multivariate analysis of radiomic features revealed that A_lbp-2D_firstorder_90Percentile (P=0.010, HR: 0.717, 95% CI: 0.557-0.924), A_log-sigma-1-0-mm-3D_ngtdm_Busyness (P=0.010, HR: 1.418, 95% CI: 1.089-1.848), and A_wavelet-LHL_firstorder_Skewness (P=0.039, HR: 1.377, 95% CI: 1.016-1.867) were significantly associated with patient prognosis (Figure 7b) (Figure s2). When incorporating both the selected radiomic and clinical features into a Cox multivariate analysis, the optimal predictive factors were identified as the radiomic feature A_log-sigma-1-0-mm-3D_ngtdm_Busyness (P=0.002, HR: 1.551, 95% CI: 1.174-2.048), the inflammatory marker ΔNLR (P=0.040, HR: 1.809, 95% CI: 1.026-3.188), and the clinical feature M stage (P=0.004, HR: 2.453, 95% CI: 1.335-4.508) (Figure 7c) (Figure s3).

### Model development and evaluation

**Figure 8:**
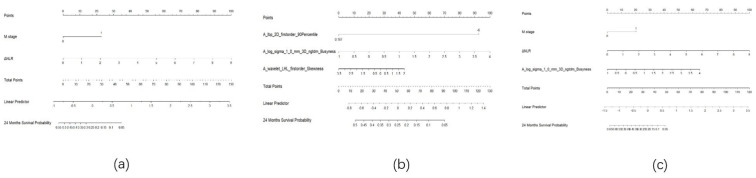
** Nomogram.** Note: (a) Nomogram for the clinical model; (b) Nomogram for the radiomics model; (c) Nomogram for the combined model.

**Figure 9:**
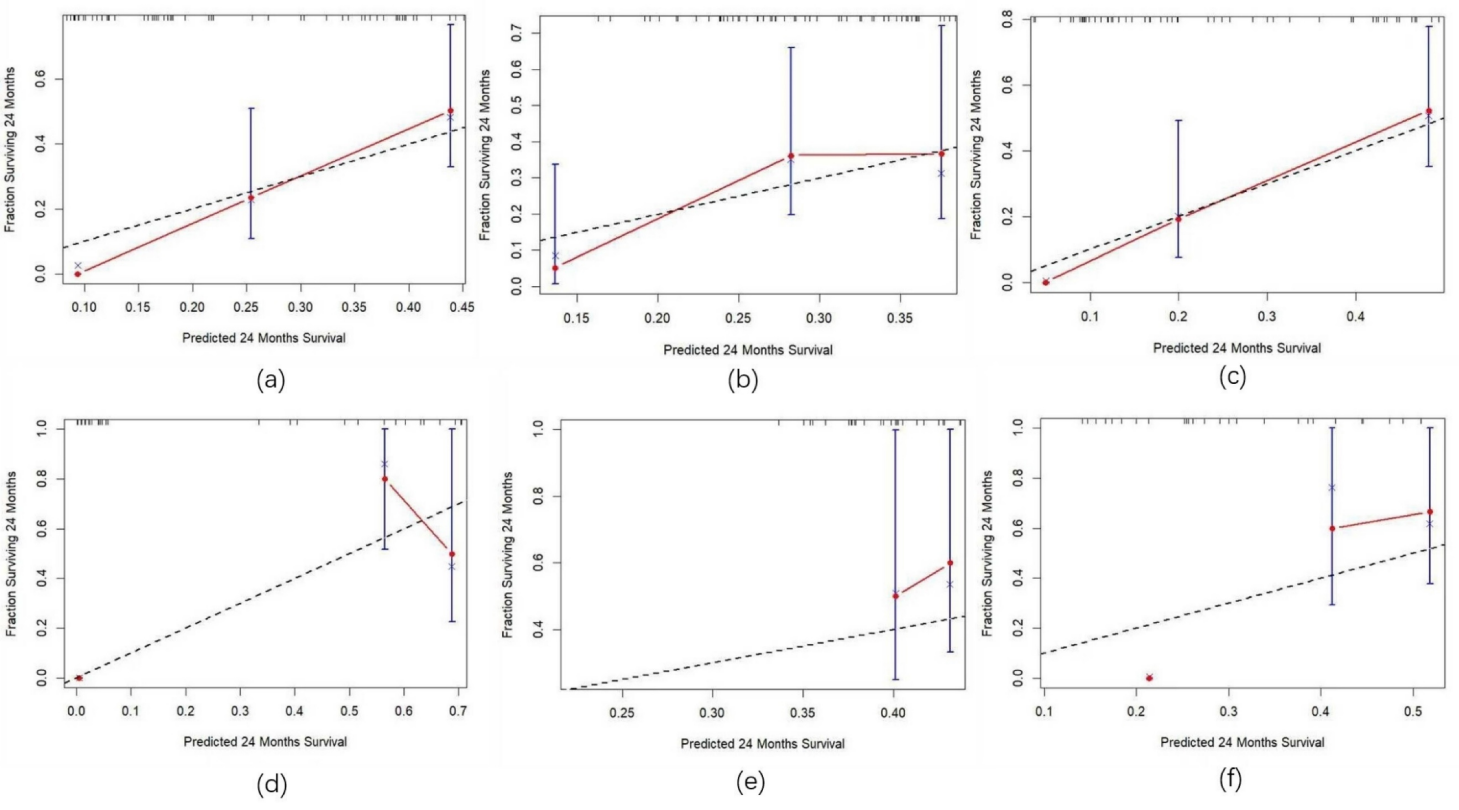
** Calibration curves for the training and validation sets.** Note: (a) Calibration curve for the clinical model in the training Set; (b) Calibration curve for the radiomics model in the training set; (c) Calibration curve for the combined model in the training set; (d) Calibration curve for the clinical model in the validation set; (e) Calibration curve for the radiomics model in the validation set; (f) Calibration curve for the combined model in the validation set.

**Figure 10:**
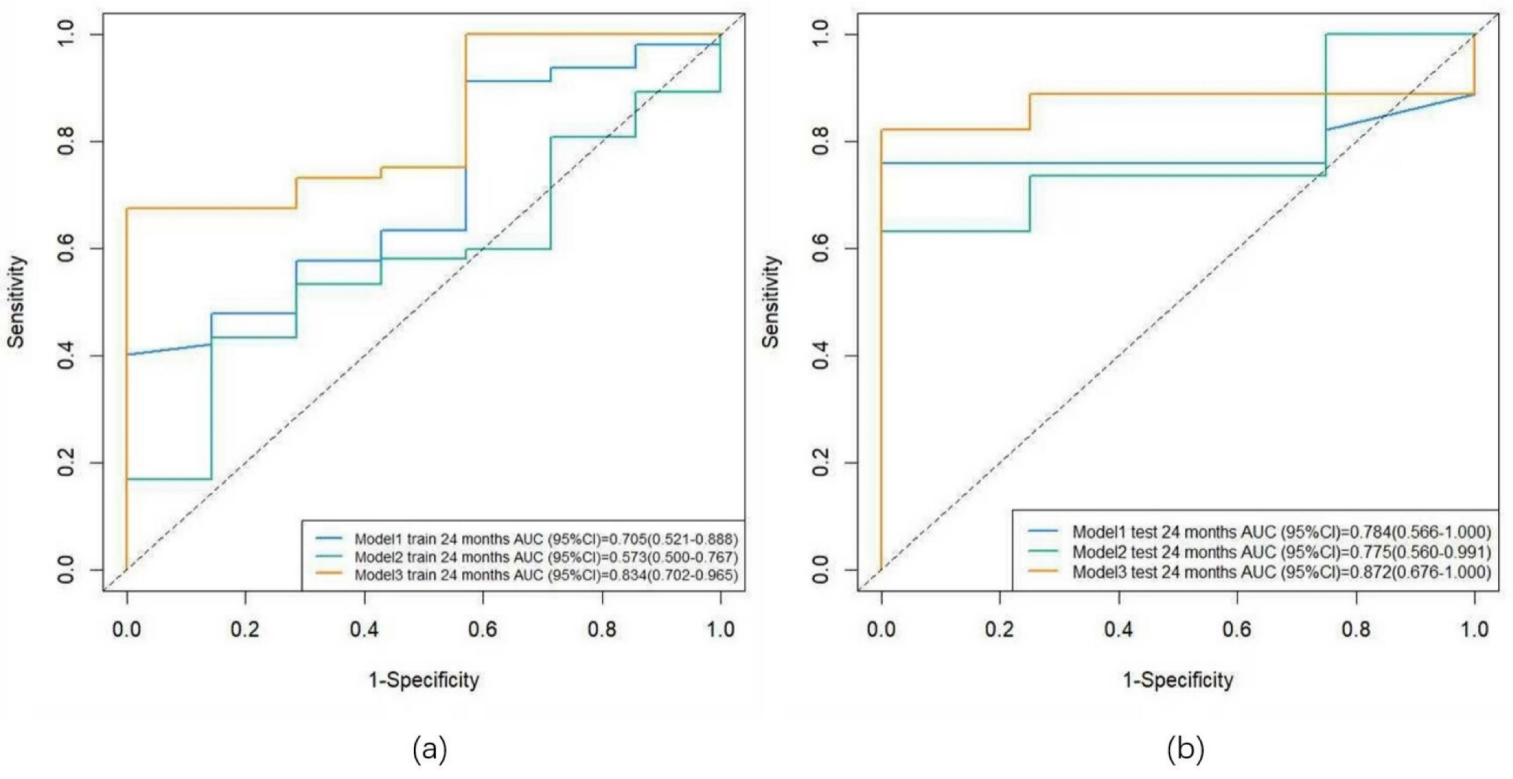
** ROC curves for the training and validation sets.** Note: (a) ROC curves for the training set; (b) ROC curves for the validation set. Model 1: clinical model; Model 2: radiomics model; Model 3: combined model.

Three predictive models were constructed based on clinical features, radiomic features, and a combination of clinical and radiomic features from Cox multivariate analysis to predict the 2-year PFS rate for inoperable ESCC patients receiving PD-1 inhibitors combined with concurrent chemoradiotherapy (Figure 8) (Figure s4, Figure s5, Figure s6). The C-index for the three models was calculated to evaluate the consistency between nomogram predictions and recorded survival outcomes. Results indicated that in the training cohort, the C-indexes for the clinical model, radiomic model, and combined model were 0.649, 0.646, and 0.648, respectively (Figure 9a, 9b, 9c). In the validation cohort, the C-indexes for these models were 0.803, 0.656, and 0.864, respectively (Figure 9d, 9e, 9f). The prediction results of all three models showed high consistency with the recorded actual survival outcomes in both cohorts. ROC curve analysis revealed that in the training cohort, the clinical model had an AUC of 0.705 (95% CI: 0.521-0.888), the radiomic model had an AUC of 0.573 (95% CI: 0.500-0.767), and the combined model had an AUC of 0.834 (95% CI: 0.702-0.965) (Figure 10a). In the validation cohort, the clinical model had an AUC of 0.784 (95% CI: 0.566-1.000), the radiomic model had an AUC of 0.775 (95% CI: 0.560-0.991), and the combined model had an AUC of 0.872 (95% CI: 0.676-1.000) (Figure 10b). The combined model demonstrated the best predictive value for the 2-year PFS rate in both the training and validation cohorts.

## Discussion

In recent years, radiomics has provided a significant complement to traditional biomarkers by identifying subtle differences in the tumor microenvironment and genomic heterogeneity, which aid in distinguishing patient responses to immunotherapy and assist in clinical decision-making 10. Additionally, various cellular components in peripheral blood play crucial roles in tumor cell proliferation, metastasis, and immune evasion 21, 22. Inflammatory markers based on peripheral blood cell components are vital biomarkers linking the tumor stroma microenvironment with immune response to therapy, and they are also important in differentiating patient responses to immunotherapy 23. However, at this stage, most radiomics studies focus on fitting analyses of extracted features, specifically calculating Rad-score values to predict disease outcomes or response to treatment. While these results support the potential of radiomics in improving patient stratification, they often lack biological interpretability. Therefore, this study established a combined predictive model by integrating radiomic features with peripheral blood inflammatory markers, aiming to provide a more interpretable basis for risk stratification in ESCC patients undergoing PD-1 inhibitor combined with concurrent chemoradiotherapy.

In this study, we utilized machine learning techniques to integrate radiomic features with blood inflammatory markers to construct predictive models. The final combined model's Cox analysis revealed that the texture feature NGTDM-busyness, the inflammatory marker ΔNLR, and the clinical feature M stage were significantly associated with patient prognosis. The combined model demonstrated optimal predictive value for 2-year progression-free survival in both the training and validation cohorts, with AUC values of 0.834 and 0.872, respectively. The C-index indicated a good consistency between the predicted results and the recorded survival outcomes. With the widespread application of machine learning and deep learning in clinical research, previous studies have combined radiomics with clinical features to establish several well-performing models. Wang *et al.* 24 utilized machine learning to predict pathological complete response in resectable ESCC patients receiving neoadjuvant immunotherapy, and the combined model demonstrated the best predictive value. Jiang *et al.* 25 employed deep learning to investigate the relationship between radiomics and immune therapy response in 321 patients with advanced gastric cancer. The combined model, after fitting pathological features and radiomic data, showed good predictive value for patient PFS. These findings align with our study, indicating that integrating radiomic and clinical features can provide a more precise basis for patient prognostic stratification. Deep learning, which combines feature extraction and model evaluation, can automate repetitive learning and training processes, significantly reducing manual effort. However, it carries the risk of overfitting and has issues with model interpretability, necessitating optimized evaluation systems to enhance its clinical applicability. In our study, the machine learning method LASSO COX utilized a penalty function to shrink the regression coefficients of the selected radiomic features. The core mechanism involves introducing an L1 norm penalty term, which constrains the regression coefficients based on traditional least squares regression, compressing some coefficients to zero. This process enables feature selection and model simplification. Subsequently, we conducted Pearson correlation analyses among these features. For feature pairs with an absolute correlation coefficient exceeding 0.9, one feature was randomly eliminated. This process was repeated iteratively until all feature correlations were below 0.9. This approach effectively reduced multicollinearity among features, enhancing the model's generalization ability and predictive performance. Furthermore, we employed a 10-fold cross-validation method during model construction. This method divided the dataset into 10 randomly selected, approximately equal-sized subsets, using one subset as the test set and the remaining nine as the training set in each iteration. This validated the model's generalizability and minimized overfitting. Therefore, the model established in this study can serve as a decision support tool for prognosis prediction and treatment planning, aiding in identifying patients likely to achieve sustained clinical benefit from immunotherapy and preventing premature discontinuation or delayed modification of ineffective immunotherapy regimens.

NGTDM features quantify the difference between the gray level of a voxel and the average gray level of its neighboring voxels within a specified distance. The primary features calculated from NGTDM include coarseness, busyness, contrast, complexity, and texture strength 26. In this study, NGTDM-busyness was identified as an independent risk factor for ESCC patients undergoing PD-1 inhibitor combined with concurrent chemoradiotherapy. Patients with favorable prognoses exhibited lower busyness in NGTDM texture features before treatment. Busyness reflects the spatial frequency of intensity variations; high busyness indicates a higher spatial frequency of intensity changes within the lesion 27.

This feature is indicative of tumor heterogeneity and aggressiveness, with higher busyness suggesting greater heterogeneity and invasiveness of the tumor 28, 29. leading to a diminished ability to sustain benefits from immunotherapy. Additionally, in our study cohort, an increase in post-treatment NLR was a predictor of poor prognosis. NLR is calculated by the ratio of circulating neutrophils to lymphocytes. A high neutrophil count is associated with extracellular matrix remodeling, promotion of angiogenesis, and tumor progression 30-32. Lymphocyte infiltration into tumors is considered an anti-tumor immune response associated with improved survival 33. Lymphopenia, commonly observed in advanced cancer, may result in weakened and insufficient immune responses 34. Early reports indicate that a reduction in serum lymphocyte count can accelerate the progression of tumor cells 35, 36. Consequently, the increase in neutrophils and the decrease in lymphocytes after treatment ultimately lead to a poorer prognosis for patients.

However, as a single-center study, this research is limited by a small sample size, lack of external validation, and inconsistencies in immunotherapy regimens due to its retrospective design, which may affect the application and generalizability of our model. To optimize our model and develop more robust and clinically applicable predictive tools, multicenter, large-sample, prospective, randomized controlled trials are needed.

In conclusion, this study confirms the importance of radiomic assessment and the detection of changes in inflammatory markers in the diagnosis and follow-up of cancer patients. The effectiveness of antitumor therapy is not solely reflected in changes in tumor size. More specifically, radiomics and inflammatory markers can reveal early changes in the internal structural characteristics of tumors, thereby evaluating the efficacy of immunotherapy and providing critical information for adjusting treatment plans, which is essential for prolonging survival.

## Conclusion

The texture feature NGTDM-busyness, the inflammatory marker ΔNLR, and the clinical characteristic M stage are independent prognostic factors for patients with unresectable ESCC receiving PD-1 inhibitor combined with concurrent chemoradiotherapy. The combined model, integrating radiomic features and peripheral blood inflammatory markers, demonstrated good performance in predicting the 2-year PFS rate in these patients.

## Supplementary Material

Supplementary figures.

## Figures and Tables

**Table 1:** Baseline clinical characteristics of patients

	Training set (n=74)		Verification set (n=31)		*P value*
**Gender**					0.363
Male	57 (77.0%)		27 (87.1%)		
Female	17 (23.0%)		4 (12.9%)		
**Age**					0.795
<60	58 (78.4%)		25 (80.6%)		
≥60	16 (21.6%)		6 (19.4%)		
**Tumor location**					0.208
Cervical	1 (1.4%)		0 (0.0%)		
Upper	6 (8.1%)		7 (22.6%)		
Middle	27 (36.5%)		10 (32.3%)		
Lower	40 (54.1%)		14 (45.2%)		
**Differentiation**					0.874
Well	7 (9.5%)		2 (6.5%)		
middle	43 (58.1%)		19 (61.3%)		
Poorly	24 (32.4%)		10 (32.2%)		
**ECOG PS**					0.953
0	22 (29.7%)		9 (29.0%)		
1	36 (48.6%)		16 (51.6%)		
2	16 (21.6%)		6 (19.4%)		
**Combined positive score**					0.351
CPS<10	48 (64.9%)		23 (74.2%)		
CPS≥10	26 (35.1%)		8 (25.8%)		
**Therapeutic model**					0.457
ICI-CCRT	11 (14.9%)		7 (22.6%)		
CCRT-ICI	11 (14.9%)		7 (22.6%)		
ICI-CCRT-ICI	16 (21.6%)		4 (12.9%)		
ICI-SCRT	36 (48.6%)		13 (41.9%)		
**Radiotherapy technology**					0.406
IMRT	40 (54.1%)		14 (45.2%)		
3D-CRT	34 (45.9%)		17 (54.8%)		
**Chemotherapy plan**					0.890
TP	65 (87.8%)		28 (90.3%)		
DP	5 (6.8%)		1 (3.2%)		
PF	4 (5.4%)		2 (6.5%)		
**T stage**					0.066
T2	2 (2.7%)		4 (29.1%)		
T3	48 (64.9%)		21 (67.7%)		
T4	24 (32.4%)		6 (3.2%)		
**N stage**					0.462
N1	16 (21.6%)		7 (23.3%)		
N2	30 (40.5%)		16 (51.6%)		
N3	28 (37.8%)		8 (25.8%)		
**M stage**					0.541
M0	43 (58.1%)		16 (51.6%)		
M1	31 (41.9%)		15 48.4%)		

Abbreviations: **ICI-CCRT:** Immune Checkpoint Inhibitor-Induced Chemoradiotherapy; **CCRT-ICI:** Concurrent Chemoradiotherapy with Immune Checkpoint Inhibitor; **ICI-CCRT-ICI:** Immune Checkpoint Inhibitor-Induced Chemoradiotherapy with Consolidation Immune Checkpoint Inhibitor; **ICI-SCRT:** Immune Checkpoint Inhibitor with Sequential Chemoradiotherapy

**Table 2:** Analysis of differences in inflammatory markers between training and validation sets

	Training set (n=74)	Verification set (n=31)	*P value*
**Pre-NLR**	2.84(1.90,3.84)	3.75(2.42,4.91)	0.047
**Pre-PLR**	139.20(104.64,184.35)	153.69(118.60,190.32)	0.246
**Pre-MLR**	0.34(0.25,0.46)	0.37±0.17	0.883
**Pre-SII**	598.25(366.93,830.12)	730.60(465.68,936.64)	0.106
**Pre-PNI**	68.31(54.82,82.35)	68.03±25.91	0.560
**Post-NLR**	2.52(1.60,4.07)	3.95±1.79	0.005
**Post-PLR**	127.85(97.11,177.88)	156.91(117.17,216.41)	0.092
**Post-MLR**	0.35(0.25,0.53)	0.36(0.28,0.59)	0.723
**Post-SII**	468.31(274.27,841.98)	657.82±327.19	0.126
**Post-PNI**	65.01±26.48	55.69±25.45	0.100
**ΔNLR**	1.07(0.55,1.40)	1.16±0.46	0.415
**ΔPLR**	0.96(0.72,1.20)	0.92(0.65,1.32)	0.911
**ΔMLR**	1.13(0.78,1.72)	1.30±0.72	0.850
**ΔSII**	0.85(0.54,1.35)	0.89±0.45	0.741
**ΔPNI**	0.94(0.68,1.22)	0.86(0.56,1.11)	0.279

Note: **pre-**: before treatment, **post-**: after treatment. **Δ:** the ratio between the corresponding time points and the markers pre-treatment.

**Table 3:** Six radiomic features selected by LASSO-Cox

	Training set	Verification set	*P value*
**LFOP**	0.167(n=72), -6(n=2)	0.167(n=27), -6(n=4)	0.111
**LSGS**	-0.17(-0.72,0.67)	0.13(-1.12,0.65)	0.883
**LSNB**	-0.38(-0.57,0.19)	-0.25(-0.49,0.20)	0.354
**WHGG**	-0.37(-0.49, -0.02)	-0.39(-0.53, -0.24)	0.325
**WLFS**	0.00±1.01	0.21±1.07	0.331
**WLGH**	0.00±1.01	-0.21(-0.71,0.52)	0.650

Note: LFOP, A_lbp-2D_firstorder_90Percentile; LSGS, A_log-sigma-1-0-3D_glszm_SmallAreaLowGrayLevelEmphasis; LSNB, A_log-sigma-1-0-mm-3D_ngtdm_Busyness; WHGG, A_wavelet-HHH_glrlm_GrayLevelNonUniformityNormalized; WLFS, A_wavelet-LHL_firstorder_Skewness; WLGH, A_wavelet-LHL_glszm_HighGrayLevelZoneEmphasis

**Table 4:** Univariate and multivariate COX analysis for the clinical model

	Univariate analysis			Multiplicity analysis		
	*P value*	HR	95%CI	*P value*	HR	95%CI
**Gender**	0.238	1.545	0.750~3.186			
**Age**	0.701	0.994	0.967~1.023			
**Tumor location**	0.179	0.969	0.337~2.783			
**Differentiation**	0.826	1.052	0.669~1.656			
**ECOG PS**	0.095	0.711	0.476~1.061			
**Combined positive score**	0.078	0.574	0.309~1.065			
**Therapeutic model**	0.358	1.141	0.861~1.510			
**Radiotherapy technology**	0.108	0.626	0.354~1.109			
**Chemotherapy plan**	0.342	0.500	0.196~1.278			
**T stage**	0.066	1.872	0.253~13.834			
**N stage**	0.057	2.649	1.121~6.261			
**M stage**	**0.005**	2.224	1.277~3.943	**0.008**	2.166	1.222~3.840
**Pre-NLR**	0.057	0.853	0.724~1.005			
**Pre-PLR**	0.805	0.999	0.995~1.004			
**Pre-MLR**	0.525	0.572	0.102~3.204			
**Pre-SII**	0.104	1.000	0.999~1.000			
**Pre-PNI**	0.192	1.008	0.996~1.021			
**Post-NLR**	0.145	1.099	0.968~1.247			
**Post-PLR**	0.509	1.001	0.998~1.004			
**Post-MLR**	0.809	1.073	0.605~1.904			
**Post-SII**	0.094	1.000	1.000~1.001			
**Post-PNI**	0.301	0.994	0.984~1.005			
**ΔNLR**	**0.001**	1.500	1.176~1.913	**0.024**	1.934	1.090~3.431
**ΔPLR**	**0.027**	1.311	1.031~1.667	0.904	1.033	0.617~1.730
**ΔMLR**	0.642	1.046	0.867~1.261			
**ΔSII**	**0.012**	1.319	1.063~1.638	0.394	0.751	0.390~1.448
**ΔPNI**	0.228	0.682	0.365~1.271			

Abbreviations: **CI:** confidence interval; **HR:** hazard ratio.
