# Supplementary material for: A machine learning model utilizing CT radiomics features and peripheral blood inflammatory markers predicts the prognosis of patients with unresectable esophageal squamous cell carcinoma undergoing PD-1 inhibitor combined with concurrent chemoradiotherapy
Source: J Cancer. 2025 Mar 3;16(6):2001–14. doi: 10.7150/jca.105171 (PMC11905411; doi:10.7150/jca.105171)
Supplement: Supplementary file 1 — Supplementary figures. [file jcav16p2001s1.pdf]

Supplementary Material

Figure.S1 Forest Plot of Cox Multivariate Analysis for the Clinical Model;

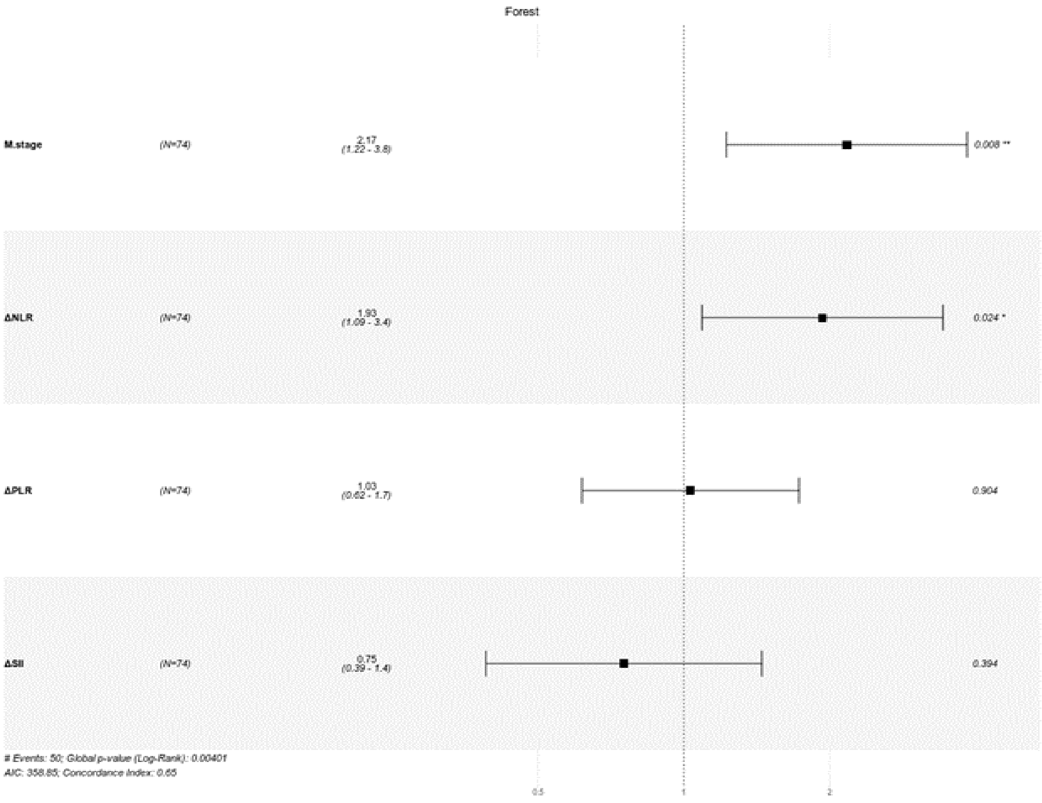

Figure. S2 Forest Plot of Cox Multivariate Analysis for the Radiomics Model;

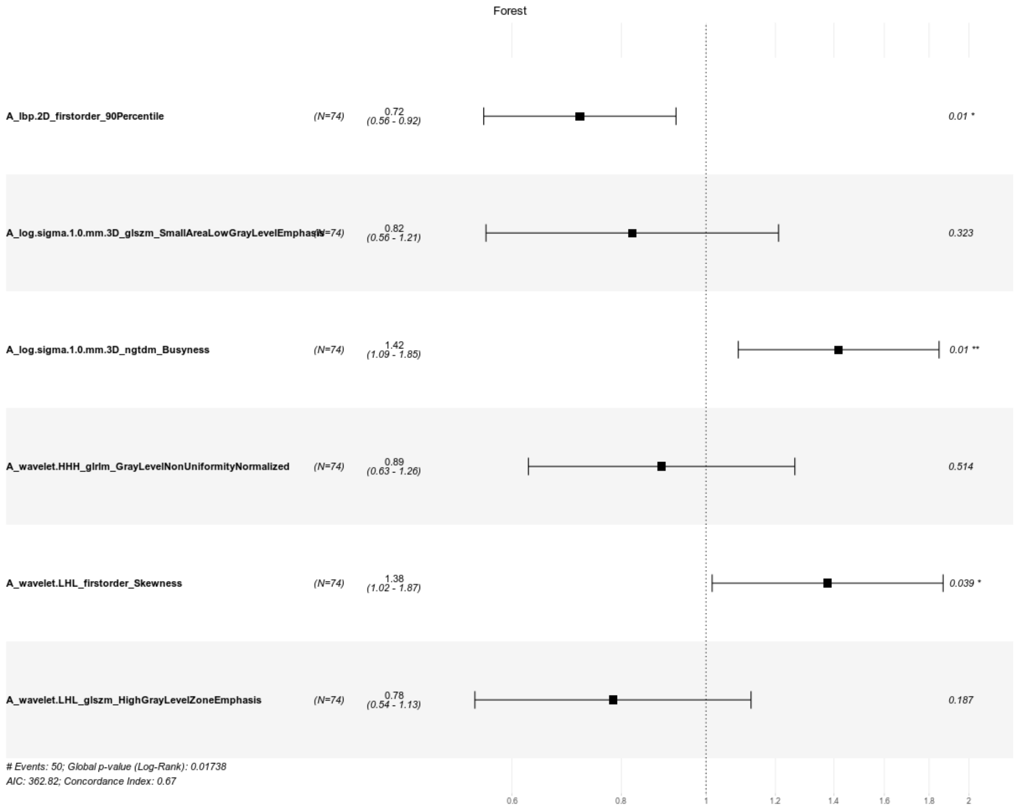

Figure. S3 Forest Plot of Cox Multivariate Analysis for the Combined Model

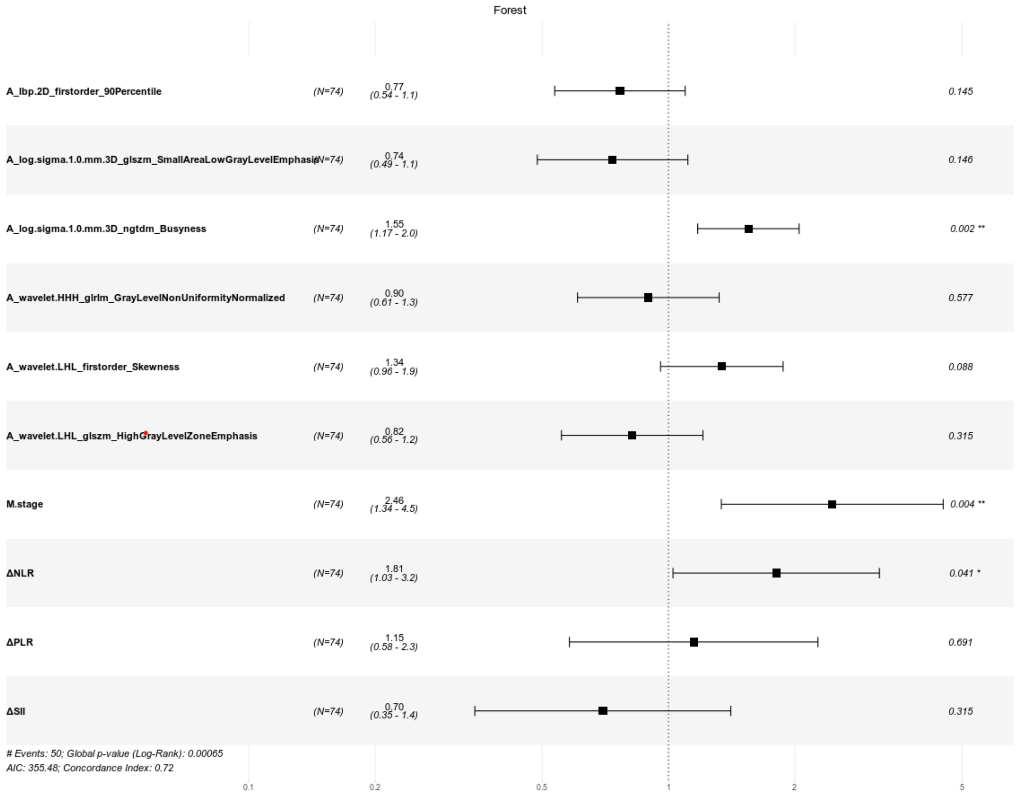

Figure. S4 Nomogram for the Clinical Model;

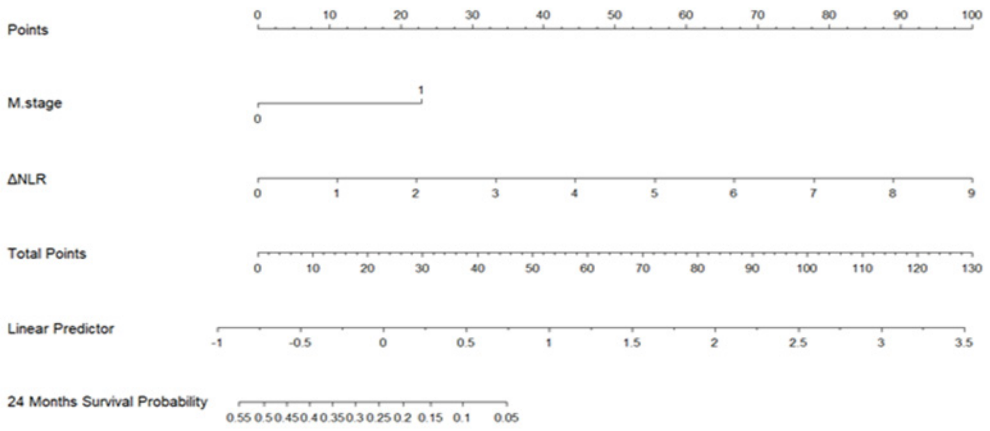

Figure. S5 Nomogram for the Radiomics Model;

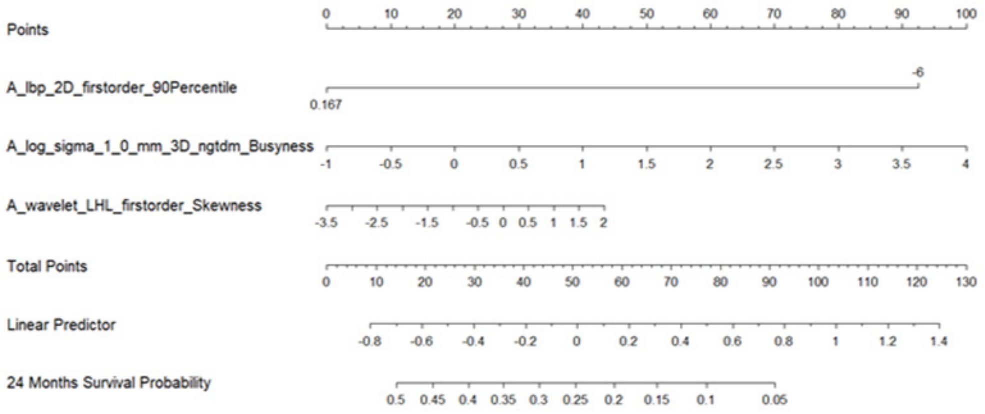

Figure. S6 Nomogram for the Combined Model.

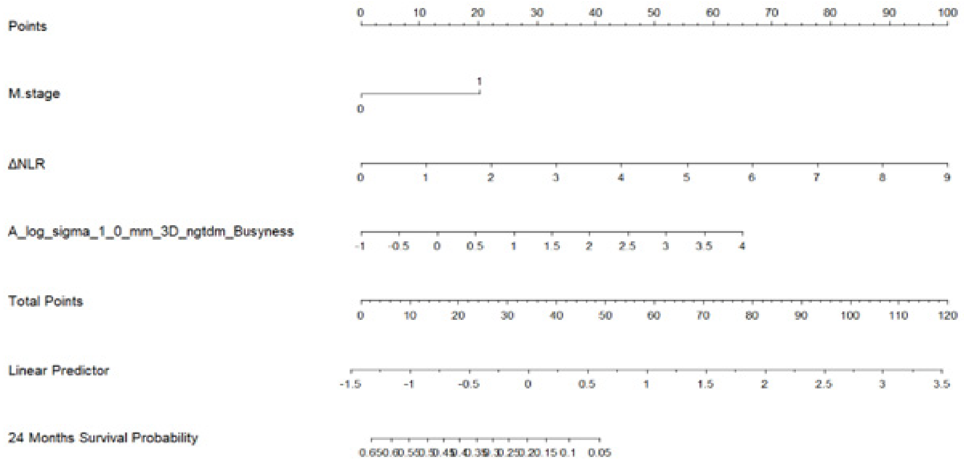

Points
0 10 20 30 40 50 60 70 80 90 100
M.stage
0 1
ΔNLR
0 1 2 3 4 5 6 7 8 9
A_log_sigma_1_0_mm_3D_ngtdm_Busyness
-1 -0.5 0 0.5 1 1.5 2 2.5 3 3.5 4
Total Points
0 10 20 30 40 50 60 70 80 90 100 110 120
Linear Predictor
-1.5 -1 -0.5 0 0.5 1 1.5 2 2.5 3 3.5
24 Months Survival Probability
0.1 0.05
